# Neonatal End-of-Life Decision Making: The Possible Behavior of Greek Physicians, Midwives, and Nurses in Clinical Scenarios

**DOI:** 10.3390/ijerph18083938

**Published:** 2021-04-09

**Authors:** Maria Dagla, Vasiliki Petousi, Antonios Poulios

**Affiliations:** 1 Department of Midwifery, University of West Attica, 12243 Athens, Greece; 2 Department of Sociology, University of Crete, 74100 Crete, Greece; 3 Department of Psychology, National Kapodestrian University, 10679 Athens, Greece; antpls@yahoo.gr

**Keywords:** end-of-life decision, neonatal care, NICU, intensive care, bioethical dilemmas

## Abstract

Background: This study investigates the acceptability, bioethical justification, and determinants of the provision of intensive care to extremely preterm or ill neonates among healthcare professionals serving in NICUs in Greek hospitals. Methods: Healthcare professionals (71 physicians, 98 midwives, and 82 nurses) employed full-time at all public Neonatal Intensive Care Units (NICUs) (*n* = 17) in Greece were asked to report their potential behavior in three clinical scenarios. Results: The majority of healthcare professionals would start and continue intensive care to (a) an extremely preterm neonate, (b) a full-term neonate with an unfavorable prognosis, and (c) a neonate with complete phocomelia. In cases (a) and (b), midwives and nurses compared to physicians (*p* = 0.009 and *p* = 0.004 in scenarios (a) and (b), respectively) and health professionals ascribing to the quality-of-life principle compared to those ascribing to the intrinsic value of life (*p* = 0.001 and *p* = 0.01 scenarios (a) and (b) respectively), tend towards withholding or withdrawing care. Religion plays an important role in all three scenarios (*p* = 0.005, *p* = 0.017 and *p* = 0.043, respectively). Conclusions: Understanding healthcare professionals’ therapeutic intensiveness in the face of NICU ethical dilemmas can improve NICU policies, support strategies, and, consequently, the quality of neonatal intensive care.

## 1. Introduction

The rapid development of medical knowledge and technology in the field of neonatal care and the improvement of living standards over recent decades have brought about significant positive changes in the epidemiological indicators related to neonatal survival worldwide [1,2,3,4]. As a result, the limits of human viability are constantly shifting towards younger and younger gestational ages. Still, however, extremely low birth weight and severely ill neonates are expected to either die shortly after birth or develop moderate to severe neurodevelopmental problems [2,5,6,7,8,9,10]. Consequently, current outcomes concerning major neurological impairments of the above-mentioned neonates have remained unchanged [7,11,12]. 

The severe disabilities of these neonates and their very poor prognosis raise significant concerns within the international scientific community [13,14,15]. In such situations, a critical question arises: Is the goal of medical care provision achieved for these neonates? Is the provision of aggressive intensive care to the benefit of such neonates, and thus, should it be provided without any limits? Or is it to the benefit of these neonates to limit intensive care? 

The above questions and dilemmas are particularly pronounced in the treatment of three categories of neonates. The first category includes neonates that are expected to die, despite the use of aggressive intensive care. In the majority of such clinical cases, the futility of the therapeutic and mechanical support provided is evident shortly after birth. It is thus argued that intensive care should be withdrawn as pointless [16,17] and nonbeneficial. The second category includes neonates who have the potential to survive (e.g., neonates with severe cerebral hemorrhage or perinatal asphyxia) but whose quality of life is expected to be poor [18,19]. The third category of neonates includes those who are able to survive without mechanical respiratory support but have an extremely poor prognosis [18,19]. The treatment of these neonates raises bioethical dilemmas for healthcare professionals employed at delivery rooms and NICUs. 

The fact that relevant approaches and practices vary between countries [20,21,22,23,24,25,26] and are disputed among healthcare professionals [22,26,27,28,29,30] intensifies the dilemmatic nature of extremely preterm and/or ill neonates’ treatment decisions. Moreover, ethical, regulatory, and medical determinants of neonatal intensive care provision interrelate to broader bioethical questions over the beginning and end of life [20,27,31], questions that are not frequently explicitly discussed [32]. However, making neonatal treatment decisions is part of the everyday practice of healthcare professionals employed in NICUs. Consequently, it is imperative to investigate these professionals’ decision-making processes; map their views, positions, and behaviors; and discuss relevant issues at the research, scientific, institutional, and social levels. This knowledge is necessary to identify the best support strategies for healthcare professionals and parents alike in end-of-life decision making and to provide an effective intensive care plan to extremely preterm and ill neonates. It can further contribute to new guidelines and a better regulatory framework, as has happened in the past [26,33,34].

In light of the above, in the present empirical study, we investigate the acceptability, bioethical justification, and sociocultural and professional determinants of the provision of aggressive intensive care to extremely preterm or ill neonates among healthcare professionals serving in NICUs in Greek hospitals. 

## 2. Materials and Methods

### 2.1. Study Population

The research protocol of the internationally recognized EURONIC project (“Parents’ Information and Ethical Decision-Making in Neonatal Intensive Care Units: Staff Attitudes and Opinions”) [35] was applied for the design of a large research project implemented in Greece detailed in Daglas and Petousi [36] and summarized here. All healthcare professionals (*n* = 495) who were employed at all public (including University and non-University) hospitals with NICUs (*n* = 17) in Greece were recruited. Of those, 251 (71 physicians, 98 midwives, 82 nurses) agreed to participate, thus attaining a study response rate of 50.7%. 

### 2.2. Materials

The main research tools included 2 anonymous, self-administered questionnaires: one addressed to physicians and one to other healthcare professionals working in NICUs. Both questionnaires had been previously used in the EURONIC project [37,38,39,40,41]. Before the beginning of field research and data collection, the study design and tools received the approval of the Ethics Committee and Scientific Board of every public hospital in which research took place. 

The survey included 3 clinical vignettes initially developed by de Leeuw et al. [41] in the context of the EURONIC project. Respondents were asked to report their course of action (therapeutic intensiveness) concerning the provision of intensive care to (a) an extremely preterm neonate, (b) a full-term neonate with an unfavorable prognosis due to severe perinatal asphyxia, and (c) a neonate with a severe congenital (physical) anomaly. The original scenarios, developed in English, were translated and culturally adjusted to the present study’s needs following the same processes implemented for the translation and cultural adaptation of the overall study tools (see Daglas and Petousi [36] for details). The present article reports the findings of this part of the analysis of the overall project. The three scenarios are presented in Table 1. 

In each scenario, healthcare professionals were presented with several potential clinical actions and asked to choose which they would follow in real life. The suggested clinical actions ranged from the application of limitations to aggressive practices sustaining the neonate’s life since the beginning to the provision of complete intensive care to every neonate in all clinical cases. Implementing the coding suggested by the developers of the scale [41], a value was assigned to each probable answer, and a summary score was calculated. Then, the mean of the standardized values from all the questions was calculated on a 5-point scale ranging from 0 to 4. For each scenario, the summary score represented each professional’s therapeutic intensiveness (from nontreatment choices to application of full intensive care) or otherwise their potential overall behavior, if this were an actual clinical case. Based on the coding, the higher the score, the greater the professional’s intensiveness to initiate and continue intensive and aggressive practices and procedures to keep the neonate alive. In contrast, the lower the score, the greater the professional’s intensiveness to apply limits to the neonate’s care. The summary score (therapeutic intensiveness) was subsequently used as the dependent variable in the present analysis. 

### 2.3. Statistical Analysis

The data were analyzed using SPSS version 22.0 (IBM Corp, Armonk, NY, USA). Results are presented as weighted proportions. A single-factor analysis of the participants’ demographic and occupational characteristics concerning the summary score of therapeutic intensiveness (the potential clinical behavior as a dependent variable) for each clinical scenario was performed. An independent samples *t*-test (for the independent variable having children) and one-way analysis of variance (ANOVA) (for the independent variables professional title, age, experience at NICU, importance of religion) were performed. Scheffé and Tamhane’s criteria were used when necessary. Pearson’s *r* coefficient was used to calculate the correlation between numerical variables, such as the therapeutic intensiveness score (dependent variable) for each clinical scenario and the “attitude score towards the value of human life” (independent variable). 

The “attitude score towards the value of human life” is a summary score that reflects healthcare professionals’ attitude towards the value of human life. The score is calculated based on healthcare professionals’ responses to a set of 12 statements developed by Rebagliato et al. [37] in the EURONIC project. Responses are measured on a five-point Likert scale (from “strongly agree” to “strongly disagree”), and respondents are asked to rate their attitudinal proximity to each of the statements. Attitude scores fall within a continuum between the “pro-life” approach or else the belief that human life has an intrinsic value and the “quality-of-life” approach. Those aligning with the position of the intrinsic value of human life tend to support initiation and continuance of intensive neonatal care that sustains the neonate’s life regardless of the outcome. Those aligning with the quality-of-life approach tend to consider that decisions over initiation, continuance, and intensity of neonatal care should be made after the neonate’s expected quality of life has been taken into consideration. Based on the suggested coding [37], the higher the summary score, the higher the alliance with the quality-of-life approach and, thus, the support towards applying limits to intensive neonatal care. The scale of the value of human life has been used repeatedly and successfully in various cultural contexts [37,38,42,43,44]. In the context of our research, the reliability of the scale was found to be high (Cronbach’s alpha = 0.77). Details on the translation and cultural adaptation of this scale for the Greek population have been presented elsewhere [45].

As part of the statistical analysis, three predictive models were also designed (one for each clinical scenario), using multiple regression analysis (method *enter*). In each model, healthcare professionals’ therapeutic intensiveness score (potential clinical behavior) was used as the dependent variable (numerical variable). Means of the professional title, the type of hospital, the hospital location, the number of mechanical respirators, the importance of religion, the experience in NICU, the number of hospitalized neonates, the total of healthcare professionals in NICU, and the “attitude score towards the value of human life” were used as independent variables for each scenario, separately. 

## 3. Results

### 3.1. Demographic and Professional Characteristics

The majority of participants were women (91.1%), 30–49 years of age (71.5%), had children (52.7%), considered the role of religion in their life important (72.9%), and had a professional experience in NICU below 15 years (78.5%). Participants’ characteristics are presented in Table 2. 

### 3.2. Factor Analysis of Participants’ Demographic and Occupational Characteristics and Their Therapeutic Intensiveness Score 

Responses to the first clinical scenario showed that the majority of Greek healthcare professionals (93.5%) are positive towards resuscitation and provision of intensive care to the extremely preterm neonate. After introducing the adverse prognosis condition due to the diagnosis of a massive unilateral hemorrhage, 40.2% of the healthcare professionals in our sample report that they would discontinue the provision of care. Additionally, approximately 1/3 (33.1%) of them state their intensiveness to seek the parents’ opinion and accept their decision.

Concerning the second clinical scenario (full-term neonate with severe perinatal asphyxia), 42.3% of the healthcare professionals in our sample state their therapeutic intensiveness to consult with the parents about the continuation or withholding of intensive care. On the other hand, more than 1/4 (26.2%) of healthcare professionals state that they would oppose the parents’ decision to discontinue the neonate’s mechanical respiratory support. 

In the third clinical scenario (baby with phocomelia), slightly less than half of the research participants (42.9%) state they would initiate medication treatment despite the parents’ contrary opinion. Only 1/5 of the participants would accept the parents’ decision to limit the provided care, whereas, in the event of deterioration of the neonate’s condition, the grand majority of professionals (71.9%) would not be willing to apply limits to the provided care. The above data are not presented in a table format.

Findings from the single-factor analysis of participants’ demographic and occupational characteristics and their therapeutic intensiveness score per clinical scenario are presented in Table 3. Concerning the first clinical scenario (preterm 24-week-old neonate, weighing 560 g, with no respiratory activity), our findings show that professional title (*p* = 0.009), having children (*p* = 0.01), the importance of the role of religion (variable categories were collapsed into two) (*p* = 0.005), and their attitude towards the value of human life (*p* = 0.001) significantly affect healthcare professionals’ therapeutic intensiveness (or else their reported intended course of action in such a case). Physicians (*Μ* = 1.5), those who do not have children (*Μ* = 1.3), and those who consider the role of religion important (*Μ* = 1.3) on average tend to have a higher summary score on the therapeutic intensiveness scale compared to their counterparts. Consequently, they tend to report a higher tendency to initiate and continue the intensive practices that keep the preterm neonate alive. The negative value of Pearson’s *r* (*r* = −0.3) between healthcare professionals’ therapeutic intensiveness and their “attitude score towards the value of human life” signifies that the closer participants align themselves to the “quality-of-life” approach, the less likely they will be to initiate and continue intensive care. Reversely, the more likely they will be to apply limits to a neonate’s care, such as that in this first clinical scenario (Table 3). 

Healthcare professionals’ therapeutic intensiveness in the second clinical scenario (full-term neonate with severe asphyxia at birth and abnormal encephalogram at six days after birth) appears to be significantly related to participants’ professional title (*p* = 0.004), the importance of religion in their life (*p* = 0.017) and their attitude towards the value of human life (*p* = 0.01). Physicians (*Μ* = 1.5) and those who consider the role of religion important (*Μ* = 1.4) on average tend to have a higher summary score on the therapeutic intensiveness scale compared to their counterparts. Therefore, they tend to be more likely to continue the provision of intensive care to the neonate with severe asphyxia. Similarly, to the first clinical scenario, the direction of the relation between therapeutic intensiveness and the “attitude score towards the value of human life” is negative (*r* = −0.2). Consequently, in the second clinical scenario, as well as in the first, the closer healthcare participants align themselves to the “quality-of-life” approach, the more likely they are to favor the application of limits to the intensive care provided to a neonate with conditions such as those described in the second clinical scenario (Table 3).

Professional title (*p* = 0.001) and the importance of the role of religion (*p* = 0.043) appear to be significantly related to healthcare professionals’ therapeutic intensiveness in the third clinical scenario (baby with complete phocomelia in need of antibiotic treatment). Physicians (*Μ* = 1.7) and those who consider religion to play an important role in their life (*Μ* = 1.4), compared to their counterparts, on average, tend to score higher in the therapeutic intensiveness scale. Consequently, physicians and people who place importance on religion indicate that they would continue providing intensive care to a neonate, such as that in the third clinical scenario. Participants’ attitudes towards the value of human life did not appear to significantly relate to their therapeutic intensiveness in this scenario (Table 3).

### 3.3. Multiple Regression Analysis for Variables Predicting Healthcare Professionals’ Therapeutic Intensiveness

Concerning the intended treatment of an extremely preterm neonate, a significant regression equation was found [*F*(14, 130) = 3.27, *p* < 0.001] with an *R*^2^ of 0.18 (that is, the predictor variables included in the equation explained 18% of the variance of the dependent variable). The predictor variables included in the equation were the following: (a) the importance of religion in the lives of our respondents (*β* = 0.21, t = 2.51, *p* < 0.05), (b) participants professional title (*β* = −0.31, *t* = −3.20, *p* < 0.01), (c) hospital type (*β* = 0.21, *t* = 2.26, *p* < 0.05), (d) hospital location (*β* = 0.35, *t* = 2.67, *p* < 0.01), (e) number of respirators in the NICU (*β* = −0.32, *t* = 2.19, *p* < 0.05) and (f) participants’ attitude towards the value of human life (*β* = −0.27, *t* = −3.22, *p* < 0.01) (Table 4). Based on this predictive regression model healthcare professionals who are employed in public rather than University hospitals, those who work in rural rather than urban areas, and medical doctors rather than midwives and nurses are more likely to report their intention to initiate and continue neonatal intensive care and support the life of the neonate. On the other hand, the higher the number of mechanical ventilators in a NICU, the more likely healthcare professionals are to support limits to the intensive care provided to an extremely preterm neonate, such as that described in the first clinical scenario. As expected, participants who align themselves with the position that life has an intrinsic value report their therapeutic intensiveness towards intensive neonatal care supporting the neonate’s life. Inversely, participants who align themselves with the “quality-of-life” approach report their therapeutic intensiveness towards limiting the intensive care provided to the neonate in the first clinical scenario. 

The regression equation used to estimate healthcare professionals’ therapeutic intensiveness in the context of the second clinical scenario (full-term neonate with severe asphyxia at birth and abnormal encephalogram at six days after birth) was also found to be statistically significant [*F*(14, 131) = 2.59, *p* < 0.01] with an *R*^2^ of 0.13. In other words, predictor variables explained 13% of the variance of the dependent variable. In this second clinical scenario, the predictor variables are the following: (a) participants’ length of experience a NICU (*β* = 0.31, *t* = 2.66, *p* < 0.01), (b) participants’ professional title (*β =* −0.41, *t* = −4.08, *p* < 0.001), (c) the number of mechanical ventilators available in the NICU (*β* = −0.32, *t* = 2.19, *p* < 0.05), and (d) participants’ attitude towards the value of human life (*β =* −0.21, *t* = −2.41, *p* < 0.05) (Table 4). Thus, in the second clinical scenario, participants with more extended experience in NICUs tend to be more positive towards initiating and continuing intensive neonatal care. As in the first clinical scenario, midwives and nurses are more likely to support placing limits to neonatal intensive care. Moreover, the higher the number of mechanical ventilators in a NICU, the less likely are healthcare professionals to initiate and continue intensive neonatal care. Finally, the closer a participant aligns with the principle of the intrinsic value of human life, the more likely they are to initiate and continue intensive neonatal care.

A significant regression equation [*F*(14, 151) = 2.35, *p* < 0.01] with an *R*^2^ of 0.10 was also found for the third clinical scenario (baby with complete phocomelia in need of antibiotic treatment). Predictor variables (a) participants’ professional title (*β =* −0.40, *t* = −4.13, *p* < 0.001) and (b) participants’ attitude towards the value of human life (*β =* −0.20, *t* = −2.51, *p* < 0.05) explained 10% of the variance of the dependent variable. It follows that medical doctors and healthcare professionals who align with the intrinsic value of the human life approach are more likely to report that they would initiate and continue intensive care supportive of the neonate’s life.

## 4. Discussion

The present research investigates the parameters that influence the ethical decision making of healthcare professionals serving in Greek NICUs via self-reported therapeutic intensiveness in three clinical scenarios. We implemented clinical scenarios developed in the EURONIC project and used widely in similar research to allow for cross-country and cross-culture comparisons. 

Our findings show that the vast majority of Greek healthcare professionals in our sample would initiate and continue the provision of neonatal intensive care. Greek healthcare professionals’ clear tendency towards supporting the neonate’s life contrasts their counterparts’ therapeutic intensiveness in other countries. According to de Leeuw et al. [41], in other European countries, such as France, Germany, and Spain, most healthcare professionals would initiate intensive care for the extremely preterm neonate, but they would probably withdraw it in the case of a poor prognosis. 

On the other hand, Greek healthcare professionals’ strong support towards sustaining the neonates’ life is in complete agreement with findings from other parts of our research. As reported in Daglas et al. [45], Greek NICU healthcare professionals closely align with the argument of the intrinsic value of human life, even in the case of unfavorable prognosis. Their vitalistic approach far exceeds respective findings for any other country in Europe or internationally. Strong support towards the intrinsic value of the human life approach has also been recorded in countries such as Italy, Spain, Ireland, and Turkey [37,42,43], countries that tend to hold traditional religious characteristics. Still, however, such support is far lesser than that recorded in Greece. 

In the context of the present study, Greek healthcare professionals’ support of the intrinsic value of the human life approach is verified because, for most of our participants, it appears to constitute the guiding principle of their therapeutic intensiveness in all clinical scenarios. Based on our findings, physicians, nurses, and midwives who side with the principle of human life’s intrinsic value are reluctant to place limits on neonatal intensive care. On the other hand, and although fewer in numbers, participants who prioritize quality of life report therapeutic intensiveness towards setting limits to the extent and the intensiveness of interventions. This association is in line with similar findings in other parts of our overall research [46] as well as findings of the EURONIC project for other countries [41]. Most importantly, these findings point to the importance of values in shaping healthcare professionals’ values and ethical decision making. 

Corroborating evidence towards the importance of values in the shaping of ethical decision making in end-of-life decisions in NICUs is our finding that in the first clinical scenario (extremely preterm neonate), the stronger the importance of religion in the life of healthcare professionals, the higher the likelihood they would support the neonate’s life through initiation and continuance of intensive care. The importance of religion as a parameter influencing ethical decision making in neonatal care has also been highlighted in the EURONIC project [41]. Similarly, other research has shown religious beliefs to be among the most common factors influencing cardiopulmonary resuscitation and neonatal intensive care decisions in extremely preterm neonates [47,48,49]. 

Another factor found to influence healthcare professionals’ therapeutic intensiveness significantly is their professional position. Midwives and nurses tend to support ethical limits to the provision of intensive care in extremely preterm infants or those with an unfavorable prognosis. In contrast, physicians are more likely to initiate and maintain treatment supportive of the neonate’s life. These findings are consistent with other parts of our overall research, documenting statistically significant differences in reported and intended therapeutic choices between physicians, nurses, and midwives. Moreover, they concur with similar EURONIC project findings [41]. Differences in the therapeutic choices between medical and nursing personnel appear to be consistent over time and verified through various types and tools of research [31,50,51]. 

Bucher et al. [25], for example, attribute the differences between the nursing and the medical staff to differences in education and training in medical ethics and ethical arguing and different professional responsibilities and roles in the end-of-life decision process of extremely preterm or ill neonates. Physicians are expected to provide the “expert’s” perspective, whereas nurses tend to be in more direct contact with the patients and their parents. Other demographic and personal characteristics of Greek healthcare professionals do not appear to influence their therapeutic intensiveness significantly. Similar findings have been recorded in other work based on the EURONIC project [41]. 

The present study was based on clinical vignettes and recorded hypothetical (intended) behavior. Healthcare professionals’ therapeutic intensiveness does not preclude their behavior in actual clinical cases. To that extent, this approach could be considered as a limitation of the present study. Nevertheless, even in a hypothetical scenario, healthcare professionals make decisions based on their beliefs and value system and their everyday practices and experiences in the NICU in which they are employed. Moreover, in clinical vignettes, participants consider factors that may or may not occur and would possibly affect them, pay attention to feelings they may be experiencing and predict possible outcomes [52]. In addition, the conclusions that emerge from the investigation of possible scenarios could serve as a starting point for the further development of the relevant research. However, the use of hypothetical scenarios is considered acceptable to investigate an individual’s actual behavior [53] and has been used in many studies in the past [41,52,53,54]. Clinical vignettes are regarded as particularly suited in the areas of healthcare and empirical bioethics, given their validity in descriptive and normative inquiries. 

## 5. Conclusions

This research presents important data related to the way healthcare professionals manage bioethical dilemmas and bioethical decision making in NICUs, regarding the acceptance of the limitation of intensive care in extremely preterm or sick neonates. The analysis of such data contributes to the recognition of the troubling position healthcare professionals are in, to the identification of factors influencing their decisions, and to the creation and improvement of the framework that is applied in NICUs and in Greece. It can also allow healthcare professionals working in NICUs to recognize ethical dilemmas, avoid moral distress, improve team cooperation, and, consequently, the quality of care provided. “Only when the organizational structure allows ethical dilemmas to be recognized, adequate decisions can be made” [55]. It is important for healthcare professionals to be aware of the values, beliefs, and possible practices concerning ethical decision making. These can influence the development of guidelines and laws and make it easier or harder for them to be accepted and implemented in a country [33]. Further research into healthcare professionals’ behaviors and attitudes towards such sensitive and ethically charged issues is considered necessary.

## Figures and Tables

**Table 1:** The three hypothetical scenarios.

1st scenario: Baby A is born at 24 complete weeks of gestation. No fetal distress is detected, but at birth, the baby appears pale, has a heart rate of 40 beats per minute, and has no respiratory activity. The baby’s weight is 560 g while the 1-min Apgar score is 1. The physician resuscitates the baby, and the baby is admitted to your Unit. Mechanical ventilation starts. A few days later, and while still in mechanical ventilation, the baby develops seizures, and the ultrasound brain scan shows a massive unilateral hemorrhage with initial enlargement of the ventricle. A periventricular parenchymal involvement is apparent on the same side of the brain. The parents are informed about the baby’s condition.
2nd scenario: Baby B is a full-term neonate who suffered severe asphyxia at birth and, on Day 6 after delivery, continues to depend on full mechanical ventilation. The baby is stuporous and hypotonic, with no sucking reflex and minimal response to stimulation. Seizures are difficult to control. The electroencephalogram (EEG) shows serious abnormalities. Major disability is anticipated in the event of survival. The parents are informed about the baby’s condition.
3rd scenario: Baby C is born with complete phocomelia (absence of the four limbs except for the presence of a 3 cm stump at the left shoulder). No other malformation is present. A maternofetal infection is suspected, and thus the baby requires antibiotic treatment. The parents object to any medical care for this baby. Antibiotic treatment is started, but the baby’s condition deteriorates. Baby C develops respiratory distress that requires endotracheal intubation and mechanical respiratory support.

**Table 2:** Demographic and professional characteristics of participants.

Demographic Characteristics	*n*	%
Age		
<30 years	46	21.3
30–39 years	83	37.9
>40	84	40.8
Gender		
Male	19	8.9
Female	200	91.1
Having children		
Yes	107	52.7
No	96	47.3
Importance of religion		
Extremely important	71	30.1
Important enough	101	42.8
Not very important	35	14.8
Not at all important	21	8.9
No answer	8	3.4
**Professional** **Characteristic**		
Professional title		
Physician	71	28.3
Midwife	98	39
Nurse	82	32.7
Experience at NICU		
<6 years	107	47.6
6–15 years	67	30.9
>15 years	47	21.5
Type of Hospital		
University	114	54.6
Non-University	99	45.4
Hospital location		
Urban	101	43.8
Rural	112	56.2

**Table 3:** Univariate analysis of the relationship between demographic and professional characteristics and therapeutic intensiveness (healthcare professionals declared intended behavior) expressed as the summary score from answers to three clinical scenarios per clinical scenario.

		1st Scenario			2nd Scenario			3rd Scenario	
Variables	*M*	*SD*	*p* *	*M*	*SD*	*p* *	*M*	*SD*	*p* *
Professional title			**0.009**			**0.004**			**0.001**
Physician	1.5	0.5		1.5	0.5		1.7	0.5	
Midwife	1.1	0.7		1.4	0.5		1.3	0.8	
Nurse	1.1	0.7		1.2	0.5		1.1	0.7	
Age			0.065			0.28			0.48
<30 years	1.4	0.7		1.4	0.5		1.4	0.7	
30–39 years	1.2	0.7		1.3	0.5		1.3	0.7	
>40 years	1.1	0.8		1.3	0.5		1.3	0.7	
Experience at NICU			0.193			0.111			0.157
<6 years	1.3	0.7		1.4	0.5		1.4	0.7	
6–15 years	1.2	0.7		1.2	0.5		1.2	0.7	
>15 years	1	0.7		1.4	0.5		1.4	0.7	
Having children			**0.01**			0.19			0.574
Yes	1.1	0.8		1.3	0.5		1.3	0.7	
No	1.3	0.6		1.4	0.6		1.4	0.7	
Importance of religion			**0.005**			**0.017**			**0.043**
Important	1.3	0.7		1.4	0.5		1.4	0.7	
Not Important	0.9	0.7		1.2	0.4		1.2	0.8	
Attitude score towards the value of human life	**0.001** Pearson *r* = −0.3			**0.01** Pearson *r* = −0.2			0.066 Pearson *r* = −0,134		

* *p* value refers to the statistical significance of the association between a given variable and the mean summary score for every clinical scenario. Significant *p* values are marked in bold. Note: *M*—Mean; *SD*—Standard Deviation.

**Table 4:** Summary of multiple regression analysis for variables predicting healthcare professionals’ therapeutic intensiveness (healthcare professionals declared intended behavior) expressed as the summary score from answers to three clinical scenarios.

	1st Scenario			2nd Scenario			3rd Scenario		
Variable	*B*	*SE B*	*β*	*B*	*SE B*	*β*	*B*	*SE B*	*Β*
Professional title	−0.52	0.16	−0.31 **	−0.45	0.11	0.41 ***	−0.64	0.16	−0.40 ***
Type of Hospital	0.30	0.14	0.21 *	−0.05	0.02	−0.40 **	−0.03	0.01	−0.20 *
Hospital location	0.51	0.19	0.35 **	0.00	0.00	0.31 **			
No mechanical respirators	−0.05	0.03	−0.32 *	−0.02	0.01	−0.21 *			
Importance of religion	0.35	0.14	0.21 *	−0.45	0.11	−0.41 ***			
Attitude towards value of life	−0.04	0.01	−0.27 **						
*R* ^2^	0.18			0.13			0.10		
*F*	3.27 ***			2.59 **			2.35 **		

Note: * *p* < 0.05, ** *p* < 0.01, *** *p* < 0.001.

## Data Availability

Data are contained within the article.
